# Surface Modification to Modulate Microbial Biofilms—Applications in Dental Medicine

**DOI:** 10.3390/ma14226994

**Published:** 2021-11-18

**Authors:** Alina-Maria Holban, Catalina Farcasiu, Oana-Cella Andrei, Alexandru Mihai Grumezescu, Alexandru-Titus Farcasiu

**Affiliations:** 1 Microbiology & Immunology Department, Faculty of Biology, University of Bucharest, 77206 Bucharest, Romania; alina.m.holban@bio.unibuc.ro; 2 Research Institute of the University of Bucharest—ICUB, University of Bucharest, 050657 Bucharest, Romania; 3 “Carol Davila” University of Medicine and Pharmacy, 050474 Bucharest, Romania; catalina.farcasiu@umfcd.ro (C.F.); oana.andrei@umfcd.ro (O.-C.A.); alexandru.farcasiu@umfcd.ro (A.-T.F.); 4 Department of Science and Engineering of Oxide Materials and Nanomaterials, Faculty of Applied Chemistry and Materials Science, Politehnica University of Bucharest, 011061 Bucharest, Romania; 5 Academy of Romanian Scientists, Ilfov No. 3, 50044 Bucharest, Romania

**Keywords:** biofilm modulation, bacteria attachment, nano-modified surfaces, dental plaque, advanced materials

## Abstract

Recent progress in materials science and nanotechnology has led to the development of advanced materials with multifunctional properties. Dental medicine has benefited from the design of such materials and coatings in providing patients with tailored implants and improved materials for restorative and functional use. Such materials and coatings allow for better acceptance by the host body, promote successful implantation and determine a reduced inflammatory response after contact with the materials. Since numerous dental pathologies are influenced by the presence and activity of some pathogenic microorganisms, novel materials are needed to overcome this challenge as well. This paper aimed to reveal and discuss the most recent and innovative progress made in the field of materials surface modification in terms of microbial attachment inhibition and biofilm formation, with a direct impact on dental medicine.

## 1. Introduction

Microbial biofilms are sessile multicellular communities acting as ultra-specialized groups of cells with different behavior and roles in the respective population [1]. Biofilms are studied for both their negative (i.e., medical biofilms that are resistant to high amounts of antibiotics and biofilm infections are very difficult to eradicate [2]) and positive (i.e., biofilm growth of an industrial bacteria into bioreactor enhances the production of the biomass and/or the desired bioproduct, such as enzyme or antibiotic [3]) impacts. This social lifestyle of microorganisms allows them to better adapt to various niches and is currently being exploited for numerous benefits. Industries, such as food, pharmaceutical and waste management, use attached microorganisms to obtain useful products and technologies.

Targeting microbial biofilms could be a beneficial approach in the prevention and treatment of many pathologies which involve a microbial component. Studies reported that more than 70% of difficult-to-treat and persistent infections are produced by microorganisms growing in biofilms. Together with genetic resistance of microbial strains, causing a worldwide crisis of antibiotic inefficiency, biofilms-embedded microorganisms show a diminished susceptibility to antimicrobials, a phenomenon known as tolerance or phenotypic resistance.

The dental plaque represents one of the most investigated multi-specific biofilms. It is an efficient model for understanding biofilms formation and relations between microorganisms in vitro and in vivo [4]. Dental plaque microbiota is very diverse, comprising over 700 microbial species, as molecular studies suggest [5], and at least 200 species have been identified as beneficial and are associated with dental health [6,7]. These include *Abiotrophia defectiva*, *Streptococcus parasanguinis*, *Streptococcus mitis*, *Streptococcus oralis* and *Streptococcus sanguinis* microbial strains [6]. On the other hand, biofilm pathogens comprise up to 100 bacterial species [8], including *Streptococcus mutans, Veillonella* spp., *Lactobacillus casei*, *Lactobacillus reuteri*, *Actinomyces* spp. and even *Candida* albicans [9,10,11,12,13].

Microbial biofilms are responsible for multiple dental pathologies, as demineralization of teeth, dental caries, prolonged inflammation and even pulp necrosis, leading to pulpitis and periapical gingivitis. Biofilms localized in the supra- and subgingival areas cause inflammation and degradation of supporting periodontal fibers, leading to bone and ultimately tooth loss. Most common diseased in this respect include chronic gingivitis and around dental implants infection (peri-implantitis) [8]. Materials utilized for dental crowns and caries treatment, as well as dental implants, become rapidly colonized by microorganisms, which are able to form thick and persistent biofilms (dental plaque), especially in difficult-to-reach areas, which remain untouched during daily hygiene.

Dental biofilm bacteria may spread to other body parts, causing bacteremia and systemic disease [10]. Therefore, efficient management of dental biofilms represents one of the most important steps in preventing and treating dental diseases.

Since microbial attachment on the oral surfaces represents the first step in biofilm development, researchers have made significant progress in elucidating attachment mechanisms and modulation. In dental medicine, the design and modification of materials aiming to improve physiological activities and aesthetics currently depend on the potential of such materials and surfaces to limit microbial colonization and the development of pathogenic biofilms [7].

Surface properties are important features of dental biomaterials and they have various functions. For example, high surface energy and rough surface texture encourage the adherence of cells forming regenerating tissues, but they may also facilitate microbial adherence. The main mechanism explaining adhesion stimulation is by increasing the contact surface. While hydrophilic surfaces enhance the adherence of host cells and microorganisms, superhydrophobic surfaces prevent this phenotype both in microbial and host cells (i.e., cells of regenerating tissues) [14,15,16].

The most investigated approaches are based on direct surface interventions, such as surface topology modifications, development of coatings for surface chemistry modification, photocatalytic applications or stimuli-responsive smart materials, that are able to detect pathogens and deliver particular antimicrobial agents to inhibit their attachment or kill bacteria [17]. Another approach relies on physical modification of the materials, which indirectly impact their antimicrobial properties by light, temperature or plasma treatment, which can be used to inhibit microbial attachment and remove mature biofilms [18].

**Figure 1:**
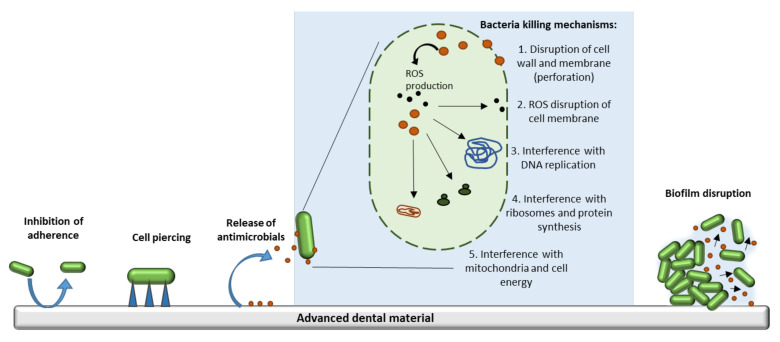
Antibacterial mechanisms in advanced dental materials. ROS = reactive oxygen species.

Depending on the approached method, the antibacterial mechanisms of newly designed dental materials are very different (Figure 1), ranging from surfaces acting as repellents by inhibiting adherence [15,19,20] to bioactive coatings [21,22,23], which specifically kill bacteria by various means, e.g., induction of membrane pores, stimulation of reactive oxygen species production [24], coupling to vital enzymes and/or DNA [25], inhibition of bacterial multiplication [26], etc.

This review paper aimed to critically discuss surface modifications in dental materials that have recently been used to prevent microbial attachment and biofilm formation.

## 2. Bacteria Attachment Factors

Bacteria prefer to grow as attached communities on a surface (i.e., dental surfaces) rather than as free-floating cultures. This growth preference offers adaptive advantages and facilitates the occurrence of difficult-to-treat infections, since attached biofilm-embedded bacteria are up to 1000 times more resistant (tolerant) to antimicrobials and host immune systems than their planktonic counterparts [27]. Surface attachment of microorganisms occurs through non-specific interactions, such as van der Waals, electrostatic forces, hydrogen bonding and hydrophobic interactions.

Attachment to dental materials is a type of solid–liquid interface attachment. Solid materials represented by natural teeth or dental materials are surrounded by a liquid environment, which is represented by saliva. The outcome of this type of adherence is governed by factors related to the bacterium on one side, but also to the solid surface, and the liquid medium (saliva), on the other side. Table 1 represents the main factors involved in bacterial attachment in a solid–liquid model.

Attachment is strongly determined by the electrical charges of the surface, hydrophobicity, hydrophilicity, wettability and topologies (i.e., roughness, geometry and other physical surface modifications) [43,44]. As most bacterial cells are negatively charged, surfaces which are also negatively charged generally repel bacteria cells and, thus, more resistant to colonization [45].

Bacteria adhere preferentially to irregular surfaces in order to maximize the bacteria–surface contact. However, this is dependent not only on the surface topographies but also on the sizes and shapes of the bacteria. Roughness, together with feature geometry, nanostructure [46], and surface physicochemistry can significantly interfere with bacterial adherence [47]. Concerning roughness, usually, a single indicator is used to describe this quantity, which is either Ra or Rq for 1D profiles or better Sa or Sq for 2D surfaces. However, these quantities only deal with the amplitude (height extension, in z, normal to the surface background plane) of roughness. There are other classes of roughness parameters, so at least one parameter describing the texture, that is the patterning due to rough features as it appears from a projection to the x–y base plane, should be considered. This deals with the spatial periodicity of the features. It is probably in this respect, that the single Sa value seem to give inconsistent results of bacterial adherence. This also depends on the spacing of the features: closely spaced ones (in the x–y plane) provide hydrophobicity and no bacterial adherence. In addition, spikiness of the rough features (associated with kurtosis, as to the height distribution, so still just an amplitude parameter) could have an effect, since bacteria usually attach to more rounded features than very sharp ones, which usually puncture them [48].

## 3. Surface Modifications in Dental Materials

There are multiple materials utilized in dental medicine, ranging from cavity varnishes, cement and restorative resins to implantable materials [49]. Depending on their chemical composition, dental materials are divided into three categories: (i) metals (i.e., gold, Co-Cr alloys, stainless steel and titanium), (ii) ceramics (i.e., Al oxide, Zr oxide, hydroxyapatite, tricalcium phosphate, bioglass and carbon–silicon) and (iii) polymers (polyethylene, polyamide, polymethylmethacrylate, polytetrafluoroethylene and polyurethane).

Surface modification in dental materials depends on the material type and intended use [50]. Virtually any material utilized in dental medicine and implantology can be surface modified [51] to alter microbial attachment and biofilm formation. In this respect, the most investigated approaches rely on four directions: (i) inhibition of attachment by surface modification; (ii) material modifications to ensure local release of antimicrobials; (iii) contact-killing; and (iv) multifunctional strategy, usually employing multiple functions (e.g., remineralizing, protein-repellent, antimicrobial, etc.) [52]. Surface changes have a great impact in the design of novel dental materials, bringing significant advantages over traditional ones. However, there are some challenges and drawbacks that need to be overcome when designing new coatings and materials for medical purpose (Table 2).

**Figure 2:**
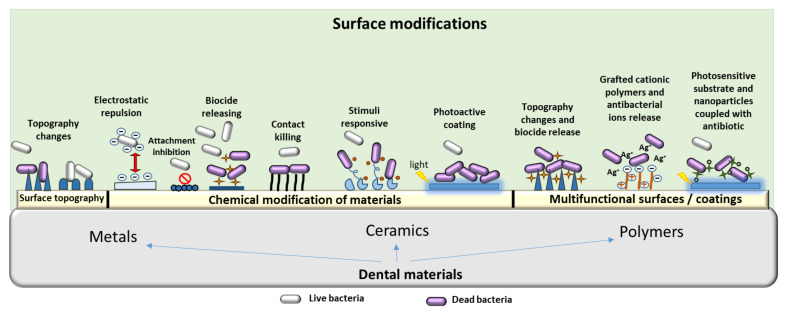
Surface modifications in dental materials investigated for their enhanced antibacterial activity. Upper part of the figure (pale green) shows different types of surface modifications which can be applied to various dental materials (i.e., metals and ceramics or polymers).

Figure 2 shows the most studied relevant modifications in dental materials to reduce microbial colonization and the development of pathogenic biofilms.

### 3.1. Titanium-Based Materials

Various studies have published the effects of topographical modifications of titanium-based materials on bacterial adherence and survival.

Lorenzetti and coworkers investigated the surface modification of titanium-based substrates in order to inhibit bacterial adherence [53]. Such substrates are frequently utilized for the design of hard tissue implants, including dental. The surface of titanium dental materials can be modified by hydrothermal treatment to synthesize nanostructured TiO_2_-anatase bioactive coatings. Titanium microasperities observed at the surface roughness (SR) scale represent a preferential site for attachment. In such conditions, individual cells interact intimately with the substrate on the valleys of the material containing high roughness. On the other hand, in TiO_2_-coated samples, the presence of nanocrystals was responsible for width reduction between the microasperities, thus adding nanoroughness features. This was translated into a decreased contact area between bacteria cells and the substrate, with up to 50% less bacterial adherence as compared to non-treated titanium material [53].

TiO_2_ nanowires obtained by hydrothermal oxidation reduce the *Pseudomonas aeruginosa* growth in the early stage of bacterial adherence, as compared with polished titanium. It was found that the titanium adherence of the dental pathogen *Porphyromonas gingivalis* was inhibited at SR levels below *R_a_* 350 nm, a roughness level generally encountered for implant collars/abutments. [54] Some studies have revealed that TiO_2_ photo-activation leads to bacteria killing in five different pathogens (*Escherichia coli*, *Pseudomonas aeruginosa*, *Staphylococcus aureus*, *Enterococcus hirae* and *Bacteroides fragilis* [55,61].

Moreover, TiO_2_ surfaces can be photoactivated upon UV irradiation, and this phenomenon has a potential bactericidal effect of titania coatings under UV light (aspect known as photokilling) [22,56,62]. TiO_2_ surfaces with photocatalytic activity can also show photo-induced super hydrophilicity, which plays an important role in the bacterial adherence process, significantly affecting surface wettability [63]. However, as a recent study reports, the increased hydrophilicity of HT-coated TiO_2_ discs obtained after UV irradiation produced no significant effects in *Escherichia coli* attachment [53].

Bacterial adherence on titanium-based surfaces with different nanotopograpy (i.e., nanotubular, nanotextured and nanorough) was also proved to be different. In recent years, several techniques for patterning material surfaces at the nanoscale [48], such as photolithography, polymer de-mixing, electron beam lithography and anodization, were developed [64].

In order to inhibit biofilm formation and show antibacterial properties, surface modification of titanium-based materials includes active antibacterial agents, such as antibiotics or nanoparticles (NPs), and repellent agents [65]. The selected bioactive agent also dictates the bacteriostatic/anti-adherence or bactericidal titanium surface character (Table 3).

In recent years, plasma treatments have been developed for titanium implants. Such treatments are intensively investigated, since they stimulate osseointegration and bone differentiation without affecting surface properties [66]. Titanium dental implants treated with a non-thermal atmospheric pressure plasma jet demonstrated significant antibacterial activity against Gram-positive and Gram-negative strains [67]. A recent in vivo study performed in Labrador dogs has revealed that Ag-plasma modification stimulates bone apposition around TiO_2_ dental implants. Such an approach has promising antibacterial properties also, considering the well-known antibacterial and anti-biofilm properties of Ag and Ag ions [60]. Because of the versatility, antibacterial efficiency and good biocompatibility with human and animal tissues, atmospheric plasma treatments are expected to be intensively explored for in vivo antimicrobial applications in the near future.

### 3.2. Ceramics

The subgingival region of dental restoration is very important for preventing gingivitis secondary caries or peri-implantitis. The SR of this region is a key factor in this respect [68], considering that bacterial adherence to restorative materials varies upon the type of material [69]. Zirconia showed the lowest bacterial adherence compared to other restorative materials, such as leucite-reinforced ceramics, noble alloys or metal-based materials, without significant differences in the SR of studied materials [70]. Hahnel et al. [71] also cast doubt over the relationship between surface properties and bacterial adherence. They found significant differences between dental ceramic classes regarding roughness but could not conclude that the SR and surface energy alone can characterize bacterial adherence mechanisms. They also evaluated the effect of a protein coating from artificial saliva, which led to a significant increase in total surface free energy (SFE), as well as in its polar and dispersed components associated with lower bacterial adherence.

Astasov-Frauenhoffer et al. [72] investigated a polymer-infiltrated Zr ceramic and three dental types of cement specimens in relation to biofilm formation. Correlations were observed between inorganic/organic composition of the materials and the polar/dispersive part of SFE. The study concluded that the formation of a biofilm composed by *Streptococcus sanguinis*, *Porphyromonas gingivalis* and *Fusobacterium nucleatum* was increased in high-wettability materials. It seems that a higher organic ratio is responsible for a lower biofilm formation ability, while a higher ceramic content results in an increased polarity and a decreased dispersity of the SFE.

Recently, Kozmos et al. investigated bacterial adherence of *S. mutans* to different dental ceramics. Interestingly, the highest bacterial adherence was observed on the yttrium stabilized tetragonal polycrystalline zirconia (Y-TZP), despite its having the lowest SR and the highest negative value of zeta potential. Correlations between those characteristics and cell adherence need to be further investigated for better material development. Surface properties, such as roughness, wettability and charge, significantly influence bacterial adherence extent [73]. Moreover, Engel et al. emphasized the importance of these characteristics next to material composition in biofilm maturation on specific restorative materials due to experimental discrepancies between the SR and thickness of the biofilm [74].

Dutra et al. [75] comprehensively reviewed the effect of finishing and polishing dental restorative materials on bacterial adherence, showing that such surface manipulations significantly affect the SR and promote a heterogeneous impact on bacterial adherence depending on the evaluated material. It is widely recognized that smoother surfaces are less likely to enhance biofilm formation. They concluded that (1) finishing procedures should always be followed by a polishing method, (2) polishing could reestablish the level of biofilm formation similar to untreated samples, (3) the range of the SR among polishing methods is wide and material dependent, (4) each dental material requires its smoothening method, (5) an SR threshold of 0.2µm did not properly predict biofilm formation for in vitro studies and (6) topographical irregularities of restorative materials had a higher impact for in vivo studies.

Intriguingly, it seems that the increased surface roughness after polishing did not increase the bacterial adherence [76,77,78], while Haralur et al. [79] reported that all polishing protocols failed to prevent bacterial adherence compared to glazed samples. While Dutra et al. tested Y-TZP ceramics, the other three studies evaluated feldspar–ceramics; therefore, the observed differences could be due to the chemical composition of the materials.

Surface properties of two translucent (5Y-ZP/8Y-ZP) and one conventional (3Y-TZP) zirconia substrates were evaluated by following a simulated clinical adjustment and intraoral finishing/polishing. The control SR was significantly higher for translucent zirconias compared to the conventional one. Material type and polishing had a statistically significant effect on SR. The four-step polishing protocol was the most efficient and exhibited the lowest surface roughness in 3Y-TZP and 5Y-TZP zirconia. The SR after this procedure was comparable to glazing for 3Y-TZP zirconia [80], in agreement with Incesu and Ianikoglu [81], who determined that the polishing performed by using an OptraFine kit (Ivoclar Vivadent AG, Schaan, Liechtenstein) determined a surface roughness of monolithic zirconia and feldspathic ceramic comparable to glazed surfaces. Scherrer et al. reported lower median roughness of zirconia and lithium disilicate ceramic than feldspathic ceramic irrespective of finishing method, emphasizing the effectiveness of chairside polishing kits, which determined an SR that was comparable to the control [82]. Poole et al. confirmed the positive correlation between the SR and colony-forming units (CFU) count of *Prevotella intermedia*, following a study on different ceramic systems [83]. Bremed et al. [84] reported significant differences in the bacterial surface coating and biofilm thickness between the various ceramic materials, following an in vivo study. HIP Y-TZP ceramic proved the lowest surface coating and biofilm thickness, while the highest values of these parameters were identified with the lithium disilicate glass–ceramic.

Çakmak et al. [85] evaluated the effect of thermocycling on resin-matrix CAD–CAM ceramics (CeraSmart, GC Corporation, Tokyo, Japan) (CS)–nanoparticle (NP)-filled resin and Lava Ultimate (LU)–resin nano-ceramic) after different surface treatments. The WCA of CAD–CAM ceramics significantly increased after conventional polishing or coating with surface sealants both before and after thermocycling. The SR was significantly affected only before thermocycling. No significant correlation was found between the surface roughness and the WCA before and after thermocycling. However, the material significantly affected the WCA and SR after thermocycling. Contreras-Guerrero et al. reported a significant correlation between the SR of ceramics’ or composites’ use of *S. mutans* biofilm formation. The samples of ceramic restorative materials found under the name IPS E-Max CAD/CAM had the lowest SR and the lowest CFU value [86,87,88].

Fluor-apatite glass–ceramics disks were coated using plasma-enhanced chemical vapor-deposited SiC to improve material’s antibacterial properties. The ceramic coating exhibited a film coverage of 19%. On the other side, uncoated samples revealed a significantly higher coverage (of 91%). The SiC coating presented bactericidal activity against *S. mutans* and *S. sanguinis* after 24 h of culture. The SiC coating produced no obvious cytotoxicity on human periodontal ligament fibroblasts. The coating slightly reduced the SR and significantly reduced the WCA [89].

Sang et al. investigated interactions between dental materials’ surface, salivary pellicle and the oral colonizer *Streptococcus gordonii* at the physical–chemical level. The kinetics of pellicle adsorption formed pellicle thickness on four materials (gold, stainless steel, aluminum oxide and Zr oxide) were similar as proved by real-time monitoring of pellicle adsorption. Pellicle deposition on all materials increased the surface WCA, surface energy and adherence of bacteria. Authors observed that surfaces with well-developed pellicles contained more attached bacteria as compared to surfaces without a pellicle. However, the physical–chemical properties of the dental material did not significantly alter bacteria attachments. New dental materials are expected to be designed for controlling bacterial attachment by optimizing the structure, thickness and composition of the adsorbed salivary pellicle in the near future [45].

### 3.3. Resin-Based Composite Materials (RBC)

Three antibacterial strategies for resin-based restorative materials were recently described: antimicrobial agent release, contact-dependent strategies and multifunctional strategy, each with its advantages and disadvantages regarding surface properties evaluated below in recent publications [90]. The morphological, physical and chemical properties of resin–dentin interfacial degradation depend on the components and chemistry of restorative materials [91], thus influencing secondary caries at the resin–dentin interface.

The relationship between superficial properties and bacterial adherence was thoroughly investigated. Yuan et al. reported a strong correlation of *S. mutans* adherence with an SR ranging from 0.02 to 0.80 µm and a weaker correlation with an SR ≤ 0.20 µm. On super-smooth surfaces (0.02–0.06 µm), bacterial adherence correlated positively with SFE. In conclusion [92]. Sainan et al. found a significant linear correlation between bacterial adherence forces and surface roughness. Furthermore, the SR exhibited less influence on the cariogenic strains than on the initial colonizers [93]. Derchi et al. reported only a partial correlation between the SR and bacterial adherence for direct dental composites [94]. CAD/CAM resin composite blocks are promising materials from a microbiological point of view, since they reduce biofilm formation in vitro when shear conditions are similar to in vivo ones [95].

Most of the recent studies that incorporate antibacterial additives into resin-based materials claim promising results. These bioactive compounds could be released and act specifically on the bacterial cells, determining targeted effects. Recent studies are reporting that novel dental materials could discriminate between commensal and pathogenic bacteria [96,97]. However, significant variability could be observed among such results and methodology, thus suggesting that they should be interpreted with caution. Surface modification was rarely investigated among the reviewed studies by Ibrahim et al. [98]

The addition of polyhexamethylene guanidine hydrochloride (PHMGH) inhibit biofilm formation to resin infiltrants. However, the critical physical properties of this material are not adversely influenced. It seems that resin infiltrants containing PHMGH at 1 wt% employed bactericidal and biofilm inhibition against *S. mutans*. The antibacterial activity is caused by a “contact-kill and release” mode of action. The WCA was slightly lower in modified samples, while the surface energy had the same levels in the test and control groups [99].

The incorporation of 2.5–10% mass fraction of dimethylaminododecyl methacrylate (DMADDM) improves the antibacterial effect expressed in *S. mutans* and limits demineralization of the tested resin. The SR and antibacterial ability have been preserved after one month of microbial-aging [100]. Dimethylaminohexadecyl methacrylate (DMAHDM) is another polymer with an antibacterial effect. Its addition decreased the biofilm CFU by 5–6 logs at 3% DMAHDM. In association with *rnc* deletion in *S. mutans* (which is an important gene for biofilm development), it had the greatest reaction in CFU by 8 logs [101].

DMAHDM is also a material with proved antibacterial activity, being efficient against several bacteria involved in the pathogenesis of dental caries and periodontal disease. When combining DMAHDM and 2-methacryloyloxyethyl phosphorylcholine or silver NPs the antibacterial effect is significantly improved. Moreover, the association with NPs of amorphous calcium phosphate or calcium fluoride ensures enhanced remineralization capacity. The SR was investigated in three studies by adding 3% and 5% DMAHDM; all studies demonstrated no alterations in SR, even after biofilm challenges [102].

Wang et al. proposed a very interesting association between 2-methacryloyloxyethyl phosphorylcholine (MPC) and DMAHDM, which greatly inhibited the biofilm formation. The MPC+DMAHDM composite showed a similar SR to commercial composite and had a 3-fold greater surface charge [103].

Surface treatment to provide anti-biofouling nature was developed by using the chemical reaction of MPC with the composite resin. The treated surface showed significant resistance to oral protein adsorption and bacterial adherence in simulated physiological conditions. The contact angle with air bubbles in an aqueous medium significantly decreased after treatment, thus enhancing surface’s wettability [104].

Lee et al. [105]. incorporated MPC in a pre-reacted glass-ionomer RBC. This study also reported a significant reduction of superficial protein adsorption and of the attachment of four-type bacteria and multispecies biofilm, inducing, at the same time, a decrease of the wettability (Table 4) of the modified RBC [105].

Some studies showed significant improvements of antibacterial properties in composites dental materials containing more than 1 wt% ZnO-NPs. However, the clinical advantage of these materials are questionable, mainly due to their short lifetime of observed antibacterial properties (similar CFU for modified and control samples after 1 and 4 weeks). Surface modifications were not investigated among selected studies reviewed by Arun et al. [107].

Quaternary ammonium polyethyleneimine (QA-PEI) NPs were extensively evaluated in recent years to establish their potential as antibiocidal additives in dental composite materials. QA-PEI NPs are newly developed and promising dental-material additives that show unique antibacterial traits, while preserving the mechanical properties of the materials [108]. The addition of QA-PEI NPs determined an increased value of the WCA but remained within the range of hydrophilic surfaces, without significantly modifying other physiochemical and mechanical properties of the 1 wt% QA-PEI-modified resin [106], in addition to their proven antibacterial activity [109].

The antimicrobial properties of a micro-hybrid composite resin of varying SRs were investigated in vitro in multispecies biofilms in two time-points (at one and four days). Increased SR was not proportional to bacterial adherence. While for *S. mutans* and *S. sobrinus* some significant differences were found in relation to SR, the adhesion of *A. actinomycetemcomitans* and *P. gingivalis* to composite resin was not significantly influenced by SR. Adherence of *S. mutans* and *S. sobrinus* evaluated strains increased significantly from one to four days, whereas the adherence of periodontal pathogens decreased from one to four days (*A. actinomycetemcomitans*, *p* < 0.001; *P. gingivalis*, *p* = 0.013). The authors observed a decreased adherence of cariogenic streptococci and total bacteria at SR values of around 0.15 µm. These results support the idea that periodic finishing of SR minimize the adherence of cariogenic streptococci to composite resin surfaces [110].

The influence of surface treatments of different RBC on *S. mutans* biofilm formation was also analyzed. Cazzaniga et al., revealed that the RBCs may impact distinctly on *S. mutans* biofilm formation, suggesting that material characteristics and composition play a greater role than the SR [111].

Bilgili et al. investigated the relationship between surface-related properties of four novel bulk-fill composites and the adherence of *S. mutans*/*S. mitis* onto the resin surface. The authors reported no significant difference between materials in terms of the SR, but there were significant differences regarding the WCA and regarding SFE, with its dispersion component being the major contributor. Researchers concluded that the SR of bulk-fill composite resins did not affect bacterial adherence, which increased with higher SFE values, especially for *S. mitis* [112].

Ionescu et al. reported that polishing of different RBCs caused a significant decrease of SFE. This was also associated with an increase of surface silicon and a decrease of surface carbon. It seems that the ratio of resin matrix and filler particles on the surface of resin-based composites strongly influences in vitro biofilm formation in *S. mutans* strains. The anti-biofilm mechanism is explained by minimization of resin matrix exposure, which could be useful to reduce biofilm formation on the surface of RBCs [113]. Polishing diminishes biofilm formation and improves the surface properties of direct and indirect resin-based composites [114]. *Candida* sp. biofilm formation on the evaluated materials was significantly impaired, and this effect was modulated by the type of finishing and polishing method [115].

### 3.4. Polymers

Polymers have been widely investigated in the development and fabrication of removable dentures (Table 5). Among these, polymethylmethacrylate (PMMA) performed better than all others. Nevertheless, the major disadvantage of the PMMA is represented by the bacterial colonization due to the absence of ionic charge [115,116,117] and the absence of intrinsic antibacterial activity [116]. The unfinished fitting surface of dentures presents numerous irregularities, which help the adherence of microorganisms that are difficult to remove by mechanical or chemical cleaning [118]. Studies reported that *c* adheres easily on this material, and it develops thick biofilms. Therefore, one of the most efficient approaches in preventing denture stomatitis is to reduce the initial adherence of *C. albicans* [23]. Denture stomatitis was reported to have a prevalence that ranges from 15% to 70% among patients [119]. We selected only studies presenting different methods of biofilm modulation associated with the analysis of surface modification.

The MPC polymers are well-investigated biomedical materials that are known for their significant biocompatibility and resistance to protein adsorption. MPC is safe as a biomaterial and has been investigated as an inhibitor of bacterial adherence on denture surfaces [120].

The surface of a PMMA resin denture base was successfully modified with MPC polymers by using a grafting polymerization technique. These surface changes retained antibacterial characteristics of the MPC against repetitive mechanical stress caused by friction induced by brushing [121]. The problem was to develop a procedure to bind MPC to denture base materials in a stable manner, as the previous grafting technique is expensive, complex and difficult to apply clinically. Ikeya et al. conducted an in vivo study on complete dentures that confirmed the viability of MPC in the form of 2-metahcryloyloxyethyl-4-azidobenzoate for inhibiting dental plaque formation for up to two weeks. This new monomer is photo-reactive and can polymerize with other monomers by conventional radical polymerization [120]. MPC (3%) and DMAHDM (1.5%) were mixed in a commercial acrylic resin, determining a much greater biofilm reduction than using MPC or DMAHDM alone. The acrylic resin kept its mechanical properties, while the average SR values were similar for MPC, DMAHDM, MPC+DMAHDM and control groups [122].

The addition of MPC or sulfobetaine methacrylate (SB), two zwitterionic materials, in 3D-printing PMMA determined a significant reduction in bacterial and biofilm adherence due to their protein-repellent properties. Although the mechanical properties were degraded, the reduction was minimal, and they maintained their resistance to the biofilm after hydrothermal fatigue. Both added materials decreased the WCA, which increased after thermocycling but reached lower values than control [123].

A study aiming to evaluate the microbial diversity of biofilm developed on the surface of acrylic resins modified with nanostructured silver vanadate (AgVO_3_) functionalized with silver NPs showed significant differences in relation to the microbial diversity of modified resins during the initial phase of biofilm maturation. The experiments were performed in natural saliva and showed different microbial diversity of early and mature oral biofilm developed on the modified acrylic resins. It seems the presence of AgVO_3_ itself interfere with the bacterial colonization, and this phenomenon is not dependent on the incorporation method, and it did not change the SR [124].

H2L is a new ligand obtained after the polymerization of methylmethacrylate with sulfadiazine and its Ag^+^ and Sn^2+^ at nanoscale. Studies reported that the modified denture base resin have stable thermal and physical properties. Since they show improved mechanical characteristics suitable for dental application, these materials could act as an intrinsic antifungal denture bases. The SR decreased in heat-polymerized acrylic resin after adding H2L, Ag^+^ and Sn^2+^ complexes [125].

Gad et al. [126] added zirconium dioxide and silver nanoparticles in different to the acrylic resin powder and found that the addition of zirconium dioxide nanoparticles to the denture base material in a double-layer technique decreased *Candida* sp. adherence and improved flexural strength without affecting the SR [126]. The increase in the SR can be explained by the difference in roughness between nano-ZrO2 and acrylic prosthesis base matrix, but also in the differences in the characteristics of the material in microscale and the shape of nano-ZrO_2_ or the inhomogeneous dispersion of nano-ZrO_2_ [127].

Methacrylate monomers containing metals were recently evaluated for their antifungal activity. This antimicrobial property does not interfere with the physicomechanical or optical properties of the denture base resin. Zirconium methacrylate (ZM), tin methacrylate (TM) and di-n-butyldimethacrylate-tin (DNBMT) are potential reactive agents for the fabrication of PMMA denture base resins with antimicrobial properties. The ZM, TM and DNBMT groups had higher antifungal activity against *C. albicans* and a lower SR than the control group [128].

Graphene oxide nanosheets (nGOs) and carbon nanotubes (CNTs) are carbon-based nanomaterials with proved antimicrobial effects. Their antibacterial mechanism is related mainly to direct contact bacteria killing properties [129,130,131]. Lee et al. [132] incorporated nGOs into PMMA to determine a sustained antimicrobial–adherence effects by increasing the wettability of PMMA. The addition of nGOs into PMMA roughened its surface and increased its wettability without compromising flexural strength or surface hardness. A sustained antimicrobial–adherence property manifested against *C. albicans* was observed in 2% nGOs for up to 28 days [132].

Han et al. investigated the incorporation of silver-based material (Novaron) in reinforced acrylic resins. Silanized aluminum borate whiskers (ABWs) (4 wt%) and nano-ZrO_2_ (2 wt%) were associated with PMMA to obtain nano-ZrO_2_–ABW/PMMA matrices. Then, various amounts of Novaron particles were incorporated into the obtained matrices. The modified composite did not have an adverse cytotoxic effect and determined a significant reduction of *S. mutans* and *C. albicans* biofilm [133]. The SR depends on the amount and distribution of NPs, i.e., a uniform distribution, leading to surface hydrophobicity and to a smooth surface; and a chemical bond with the PMMA chain [134].

Incorporation of pre-reacted glass-ionomer filler slightly increases the SR of denture base resin, but it reduces the adherence of *C. albicans*. Materials containing at least of the 5% filler revealed a thinner biofilm as compared to the control group. All filler groups showed hyphal forms at 3 h, with the length of the hyphae being lesser than those in the control group [135].

Using a hybrid process of plasma-based ion implantation and deposition, fluorine and silver ions were added into the PMMA resin. The obtained material proved a remarkable antibacterial activity and was associated with the development of a more hydrophobic surface. It seems that fluorine and silver dual-ion implantation and deposition can enhance antibacterial properties of novel acrylic medical and dental devices [136].

The addition of Si_3_N_4_ ceramic particles (~8% vol.) in self-curing PMMA exhibited fungicidal action against *C. albicans* while being synergic with chemoprophylaxis. Investigations proved that there was no significant loss in bulk properties of the resin. Similar morphologies were observed for the PMMA and PMMA + 8% vol. Si_3_N_4_ substrates, except for the presence of some irregularities in the ceramic particles on the surface of the latter samples, had an increase of 26% in the mean roughness of the modified resin [137].

Studies demonstrate that, when adding 25 ppm copper (Cu) NPs to denture base resins, the obtained material is significantly inhibiting biofilm formation of *C. albicans* dental strain. Although this study did not investigate surface properties, a surface modification is plausible. The antimicrobial role of Cu NPs could be substantiated with NPs piercing the microbial cell wall and stimulating the release of reactive oxygen species [138].

Poly(N-vinyl-2-pyrrolidinone)-grafted PMMA denture materials have been investigated a lot in recent years. One explanation regarding their popularity is that such materials can be repeatedly recharged with antimicrobial drugs. Sun et al. reported no physical properties and biocompatibility changes when the resins were modified by up to 7.92% of PNVP grafting. The model antimicrobial drugs were two antifungals, namely miconazole and chlorhexidine digluconate (CD). PNVP grafting significantly increased the antimicrobial substances absorption of the resulting denture materials. The released drugs showed potent antifungal and biofilm-formation effects against *Candida* sp. Grafting significantly improved the wettability of the resin surface [139].

The addition of dimethylaminohexadecyl methacrylate (DMAHDM) and chlorhexidine diacetate in a self-cured resin proved to increase antimicrobial activity against *S. mutans* or *C. albicans* significantly. The SR increased compared to control. No cytotoxic effect was recorded for this association [140].

Some studies reported that probiotics, such as *L. rhamnosus* and *L. casei*, interfere with *Candida* sp. biofilm initial formation and maturation. Neither of the probiotics affected the SR of the denture base resin, as recent studies report [141].

Chitosan NPs (ChNPs) have a widely recognized antimicrobial effect, being investigated both in bacteria and microscopic fungi. However, there is a limited information about the effects of ChNPs against *Candida* sp. biofilm on denture base surface. Recent studies showed antifungal activity of chitosan NPs against planktonic *C. albicans, C. tropicalis* and *C. krusei.* These NPs also inhibited the initial adherence and mature biofilm development in *C. albicans*. The chitosan NPs significantly reduced the CFU (colony forming units) of *Candida* spp. developed on acrylic surface containing such NPs after 5 days. These NPs reduce the SR and hardness of the material as compared to sodium hypochlorite, which is frequently used to disinfect denture [142].

Cold plasma treatment of PMMA denture base resin was recently investigated for oral medicine. This treatment could produce oxygen-containing polar hydroxyl, carbonyl and carboxyl groups at the polymer surface, thus increasing the SFE and wettability of the polymer and improving its adherence to oral tissues. Plasma modified of PMMA decreases the WCA by 1.5–2.5 times as compared to unmodified samples, while their SFE increased up to 1.5 times due to the formation of additional plasma induced oxygen-containing polar chemical groups. The plasma treated denture surface proved a good biocompatibility and less irritating effects, as compared to non-modified surfaces [143]. The wettability improvement of plasma-treated samples results from changes in surface morphology and surface chemistry [144].

Shibata et al. [145] evaluated a coating containing poly(2-methacryloyloxyethyl phosphorylcholine-co-n-butyl methacrylate) PMB which was developed for improving PMMA materials. This coating drastically reduced the ability of cariogenic bacteria, such as *S. mutans* and *S. Sobrinus*, to develop biofilms. Another study regarding the effects of MPC-polymer coating on denture base resins on the adherence of *C. albicans*, non-albicans *Candida* (NCAC) and methicillin-resistant *Staphylococcus aureus* (MRSA) was conducted by Fujiwara et al. [146]. MPC-polymer showed a limited effect against the growth of *Candida* sp. and MRSA strains evaluated, but it significantly suppressed adherence to denture resin in most of the evaluated microorganisms. The inhibition of adherence is caused by the increased hydrophobicity of the resin surface treated with 5% of MPC-polymer. The resulting surface proved to be significantly more hydrophilic and with a higher wettability. Turkcan et al. confirmed the MPC coating induced a significant increase in wettability, no differences regarding surface roughness and a significant decrease of *C. albicans* adherence [147].

Coatings based on resins prepared with a cross-linkable copolymer containing sulfobetaine methacrylamide (SBMAm) inhibited the adherence of *C. albicans*, as revealed by CFU results and SEM (scanning electron microscopy) images. Adherence inhibition expressed by the coating seems to be correlated with the wrinkle-based structures of the surfaces coated with copolymers containing more than 30% SBMAm. However, the SR was not significantly different among all groups. In conclusion, cross-linkable copolymers containing SBMAm can enhance surface hydrophilicity in denture-base resins and reduce the initial adherence of *C. albicans* [23].

A plasma coating with trimethylsilane monomer was proved to significantly reduce the adherence of *C. albicans* to denture base resin. Plasma treatment exhibited a significantly more hydrophobic surface on which *C. albicans* was found to grow less than the control group, while adherence tests showed a significantly lower adherence of the *C. albicans* strains on the coated surfaces [148].

Polyacrylic acid (PAA) and poly itaconic acid (PIA) were tested as surface treatments on conventional denture base materials (Table 4). Both acids exhibited a significant *C. albicans* growth inhibition. The incorporation of carboxylic groups by using their coatings reduced the adherence of *C. albicans* by 90%. Both acids improved the wettability of the substrates, but PAA significantly increased the roughness of both tested denture materials but had no cytotoxic effect on human cells [149].

The mechanical properties and SR of CAD–CAM PMMA resins are also improved, as compared with heat-polymerized polymethyl methacrylate resin. Conventional heat-polymerized PMMA resin proved to have higher SR values as compared to CAD–CAM PMMA resins [150]. Printable denture material was surface modified with nano silver-loaded Zr phosphate (6S-NP3) obtained from simultaneous silanization of γ-methacryloxypropyltrimethoxysilane (MPS) and grafting reaction of methyl methacrylate (MMA). This type of modification improved the material’s mechanical properties but increased the WCA of the surface. The obtained composites proved great antibacterial activities against *S. aureus* and *E. coli* [151].

Mangal et al. [152] evaluated the role of nanodiamonds (NDs) as fillers to enhance the resistance to friction and wear but also the bacterial functions of dental materials. The control specimens without ND fillers were tested against specimens with both amine-functionalized NDs (A-ND) and pure non-functionalized NDs. After the addition of 0.1 wt% ND in the PMMA-based resin for 3D printing, the mechanical properties and resistance to bacteria colonization were significantly improved. This effect seems to be dependent on NDs’ functionalization, while the SR proved no significant differences between sample groups. However, the SR increases in amine-functionalized surfaces.

Simoneti et al. [153] compared single interim crowns obtained by 3D-printing (laser stereolithography (SLA) and selective laser sintering (SLS)) and conventional methods with acrylic resin and bis-acryl resin regarding mechanical properties and biofilm formation. The SR presented significant variations when comparing the improved materials, being different before and after finishing. The conventional materials and SLA presented similar SRs, which were lower as compared to SLS value. Biofilm development showed insignificant variations on these materials.

## 4. Conclusions

This paper aimed to reveal the amount and diversity of surface modifications for improved dental materials, highlighting the approaches utilized for obtaining antimicrobial and anti-biofilm effects. The limitation of microbial attachment, which is the first step in biofilm formation, still remains a target when designing dental medicine materials. However, there is a series of challenges in designing ideal dental materials when it comes to their surface properties. Novel materials and coatings should be smooth enough to allow the attachment and proliferation of the host’s soft tissues’ attachment and proliferation, while limiting the adherence and biofilm formation of dental pathogens. Moreover, the attachment of oral microbiota on dental surfaces could be beneficial for maintaining the balance for oral health. In recent years, particular attention was given to the smart, stimuli-responsive or functional materials, which could be capable of inhibiting attachment to ensure the targeted killing of dental pathogens. However, additional studies are still necessary to reveal the intimate antibacterial mechanisms and how these will impact the management of oral diseases. Certainly, significant research on these novel materials is needed to confirm a real breakthrough in the longevity of restorative dental materials and their efficiency and potential side effects at mid- and long-term utilization.

## Figures and Tables

**Table 1:** Main Factors that Balance Bacterial Adherence to a Solid–Liquid Interface.

Categories	Factors	Effects	References
Bacterium related factors	Surface charge	Negative surface charged bacteria interacts better with positively charged surfaces; the effect is altered by high ionic strength media and can be influenced by Quorum Sensing (QS) molecules (i.e., acyl homoserine lactones (AHLs)	[28]
	Surface energy	The surface energy of bacteria is typically smaller than the surface energy of the surrounded liquid; therefore, microbial cells tend to adhere better to hydrophobic materials	[28,29]
	Shape and size	Perpendicular or parallel orientation of bacteria (i.e., rod-shaped) to a surface is possible to meet the dimensional constraints but also thermodynamic requirements	[29]
	Appendages	Ensure direct interference surface topographies, detection of surface-associated mechanical/chemical cues (i.e., fimbria and pili)	[28,30]
	Adhesins	Ensure surface colonization and facilitate cell–cell cohesion (i.e., fimbrial and non-fimbrial proteins)	[31]
	Extracellular polymeric substances	Masking of the effective topographies, participate to the conditioning film development (e.g., capsula)	[29]
	Quorum Sensing (QS) molecules	Small signaling molecules detected by bacteria cells which orchestrate the behavior of a complex microbial community (i.e., AHLs) and alter the movement of appendages, such as flagella, and the cell surface charge)	[28,32]
Liquid medium related factors	Temperature	Temperature changes reduce attachment and biofilm development (i.e., biofilm development is optimal at 30 °C for most bacteria, but significantly impaired at 60–70 °C)	[33]
	Ionic strength	Low ionic strengths inhibit bacterial adherence, while higher ionic strengths facilitate irreversible bacterial surface adherence (i.e., 0.85% saline solution is optimal environment for adherence in most bacteria)	[34]
	Viscosity	Viscous liquids impair microbial movement and attachment	[28]
	Surface tension	The high surface tension of water is not appropriate for bacteria to pierce the air–liquid interface; therefore, it is expected that high ratios of air–liquid to solid–liquid interfaces to inhibit bacterial attachment (main mechanism is related to the air entrapment)	[29,35]
	Hydrodynamics	Surface topography at the microscale can influence hydrodynamics, which, in return, impact bacterial attachment under flow conditions (motion and deformation are key parameters)	[36,37]
	pH	Local pH variations alter bacteria surface sensing, attachment and biofilm formation (i.e., alkaline pH of 7.4 is optimal for biofilm development, while at pH of 6.0, attachment and biofilm are significantly impaired)	[38]
Substratum related factors	Surface charge	Positively charged surfaces are colonized by bacteria faster	[19]
	Surface energy	Low surface energy reduces surface wettability and, thus, attachment	[19]
	Topography	(a) Roughness is the most deployed parameter, as bacterial attachment is increased with higher roughness; (b) spatial details, such as geometry, periodicity, symmetry, density or hierarchical structure of the surface characteristics, are important for bacterial adherence	[29,39,40]
	Stiffness	Increasing stiffness of hydrophobic surfaces correlates with decreasing adherence; on the other hand, high stiffness of hydrophilic surfaces increases bacterial adherence and biofilm formation	[20,41,42]
	Conditioning film	Modulate bacterial attachment by: (a) Surface properties changes of the neat material surface; (b) Surface topography changes; (c) Bacteria–surface interactions particular sites.	[21,29]

**Table 2:** Advantages and Weaknesses in Dental Material Modifications.

Material Modification	Advantages	Weaknesses	References
Surface modification	Inhibition of bacterial adherence, limitation of the pathogenic biofilm formation	May interfere with adherence of regenerating cells and tissues; also impair the adherence of commensals	[53,54,55,56]
Chemical modification to ensure drug release	Targeted antibacterial activity, controlled release of the drug	Local hypersensitivity, inflammation	[22,24,27]
Contact-killing	High efficiency in bacteria killing; rapid effects	Sometimes lacks the bacterial killing specificity, it may interfere with repair host cells and tissues	[57]
Multifunctional coatings and surfaces	Versatility, high efficiency, multiple bioactivities for a tailored therapy	Unknown mid- and long-term side effects	[44,58,59,60]

**Table 3:** Surface Modifications for Achieving Antimicrobial Properties of Titanium-based Dental Materials.

Surface Modifications	Methods	Effects	Antibacterial Mechanisms	Applications	References
Low surface energy	Plasma treatment, chemical treatment (i.e., sandblasting and/or acid etching)	Anti-adherence	Passive inhibition of bacterial adherence	Dental implants	[58]
Electrostatic repulsion	Layer-by-layer electrostatic self-assembly	Anti-adherence	Passive inhibition of bacterial adherence by using ions coats	Dental implants	[55]
Exclusion steric repulsion	Chemical grafting	Anti-adherence	Passive inhibition of bacterial adherence by using polymeric coats	Various Ti implants	[55]
Contact active bactericidal surfaces containing synthetic agents (i.e., quaternary ammonium compounds and polycations)	Polymeric coating	Quaternary ammonium compounds change the bacterial cell essential ionic balance, disturbing the cellular membrane.	The active killing of bacteria cells by direct binding to their cell membrane and interaction with the negatively charged structures.	Dental implants	[57]
Contact active bactericidal surfaces containing natural agents (i.e., antimicrobial peptides (AMPs))	Electrochemical modification, coating	AMPs (positively charged) interact with the bacterial membranes (negatively charged); involved in membrane piercing and DNA damaging	Active killing by membrane piercing due to competition with calcium and magnesium ions linked to the bacterial polysaccharides; may attach on the membrane via anionic phospholipids and phosphate groups of polysaccharides; change the bacterial membrane electrochemical gradient and the cell morphology	Ti-based dental implants	[57]
Antibiotic releasing surfaces	Chemical grafting, coating		Active killing by the specific antibacterial mechanism	Ti bioactive implants	[59]

**Table 4:** Modification of Resin-based Composites to Achieve Surface Properties to Control Bacterial Attachment and Biofilm Formation.

Methods	Materials	Surface Modifications	References
Composition modification	Polyhexamethylene guanidine hydrochloride (PHMGH)	WET increased, SFE~control	[99]
	DMADDM, DMAHDM	SR~control	[100,101,102]
	MPC+DMAHDM	SR~control, SFE increased	[103]
	MPC	WET increased	[104]
	MPC in pre-reacted glass-ionomer	WET decreased	[105]
	quaternary ammonium polyethylenimine (QA-PEI)	WET decrease (hydrophilic surface)	[106]

**Table 5:** Modification Strategy inP to Achieve Surface Properties to Control Bacterial Attachment and Biofilm Formation.

Methods	Materials	Surface Modifications	References
Composition modification	MPC, DMAHDM, MPC+DMAHDM, AgVO_3_, nanodiamonds	SR~control	[122,124,152]
	H2L + Ag^+^/Sn^2+^, Zirconium methacrylate, Tin methacrylate, di-n-butyldimethacrylate-tin	SR decreased	[125,128]
	Graphene-oxide nanosheets (nGo)	SR increased WET increased	[132]
	ZrO_2_, pre-reacted glass ionomer, Si_3_N_4_ ceramic particles, DMAHDM + chlorhexidine diacetate	SR increased	[127,135,137,140]
	Fluorine and silver ions	WET decreased	[136]
	Poly(N-vinyl-2-pyrrolidinone), SB (sulfobetaine methacrylate), MPC	WET increased	[123,139]
Surface treatments	Probiotics, chitosan	SR~control	[141,142]
	Cold plasma	WET increased SFE increased	[144]
Coatings	MPC, sulfobetaine methacrylamide	WET increased SR~control	[23,147]
	Trimethylsilane, nano silver-loaded zirconium phosphate	WET decreased	[148,151]
	Poly acrylic acid	SR increased WET increased	[149]
	Poly itaconic acid	WET increased	[149]

## Data Availability

Not applicable.
